# Supplementary material for: A gRNA-tRNA array for CRISPR-Cas9 based rapid multiplexed genome editing in Saccharomyces cerevisiae
Source: Nat Commun. 2019 Mar 5;10:1053. doi: 10.1038/s41467-019-09005-3 (PMC6400946; doi:10.1038/s41467-019-09005-3)
Supplement: Supplementary file 1 — Supplementary Information [file 41467_2019_9005_MOESM1_ESM.pdf]

Supplementary Information

A gRNA-tRNA Array for CRISPR-Cas9 Based Rapid Multiplexed Genome Editing in *Saccharomyces cerevisiae*

Zhang et al.

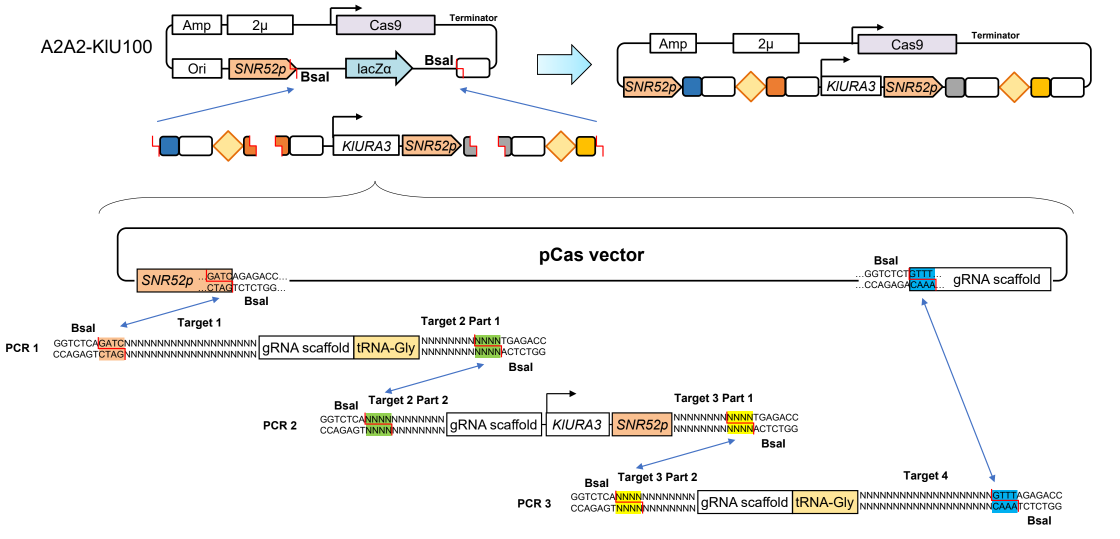

(A2A2-KlU100). The pCas vector was constructed with the lacZα sequence flanked by a *SNR52* promoter and a gRNA scaffold with BsaI cleavage site at the end of the *SNR52* promoter (GATC) and the beginning of the gRNA scaffold (GTTT). To assemble multiple gRNAs on one plasmid, the lacZα sequence is removed by BsaI digestion and replaced by PCR-generated fragments. The specific 20bp targeting sequences are designed to be included on the primers. The 4bp DNA highlighted in brown, green, yellow and blue represent the ligation site for Golden Gate reaction. The detailed primer designs are described in Supplementary table 1 and PCR template sequences are listed below. The Golden Gate assembly for other plasmids in this paper are in the same principle.

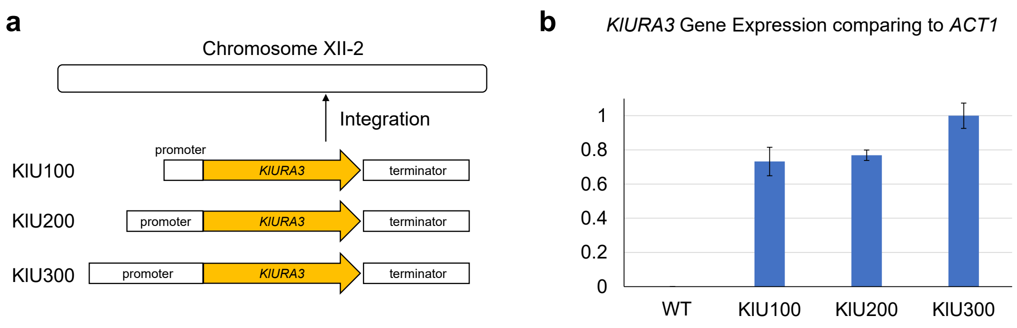

Supplementary Figure 2. Gene expression for KlURA3 with different sizes of its promoter. (**a**) Graphic representations of integration of *KlURA3* with different sizes of its promoter. (**b**) Gene expression analysis of *KlURA3* comparing to *ACT1* by real-time PCR. The error bars indicate the standard deviation of all (n=4) biological replicates. Source data are provided as a Source Data file.

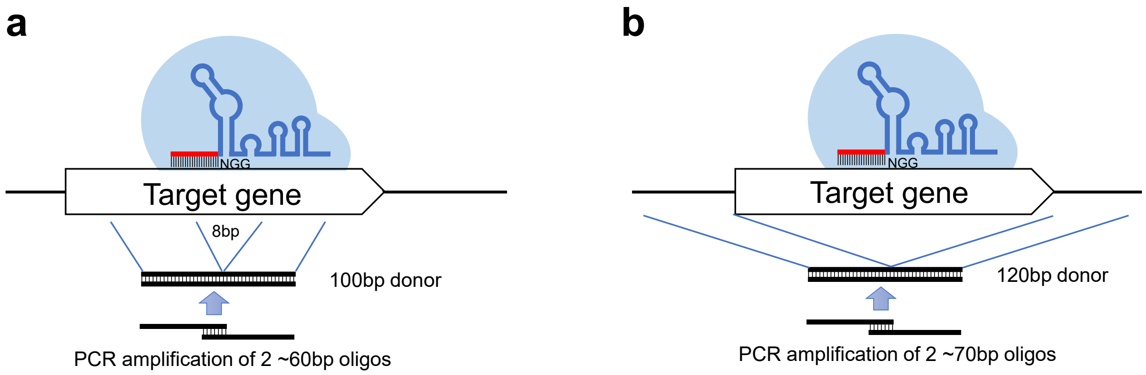

Supplementary Figure 3. Graphic representation of the PCR generation of donors for GTR-CRISPR system. Donors for repairing DSB were generated through PCR amplification of two oligonucleotide primers for (**a**) gene disruptions 100 bp and (**b**) whole ORFs deletions 120 bp. The Melting Temperatures (Tm) for primers are in the range of 52-60°C.

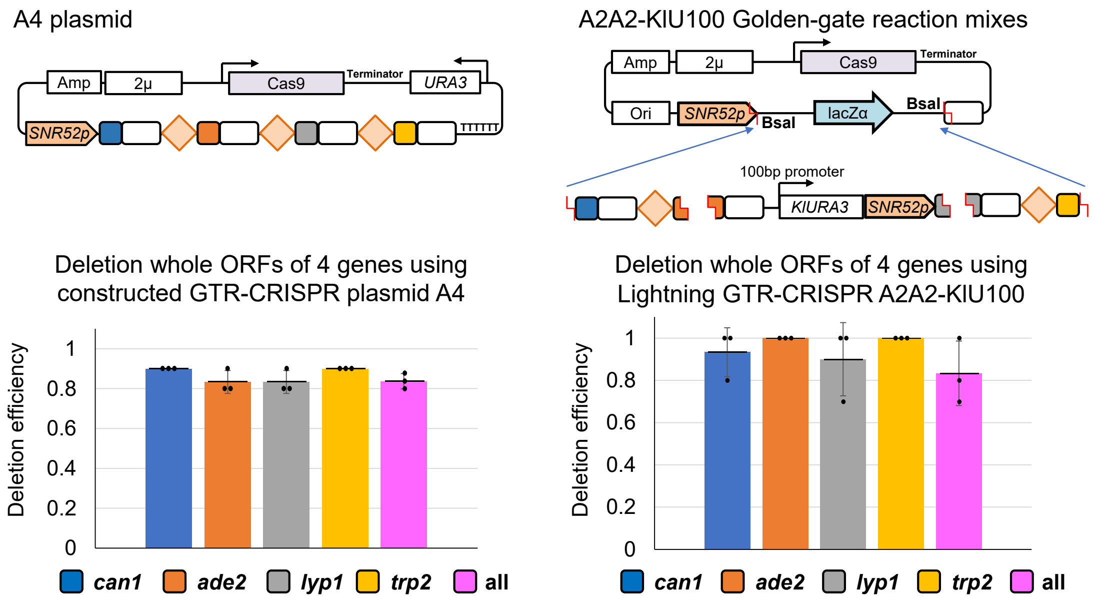

Supplementary Figure 4. GTR-CRISPR system and Lightning GTR-CRISPR system for simultaneous whole ORF deletions of 4 targets. Graphic representations and results of deleting the whole ORFs of 4 genes using constructed plasmid (GTR-CRISPR system) or Golden Gate reaction mix (Lightning GTR-CRISPR system). The data of bar charts represent mean averages of each gene or over-all disruption efficiencies. Each black dot represents the gene disruption efficiency of 10 colonies from each biological replicate, and the error bars indicate the standard deviation of all (n=3) biological replicates. Source data are provided as a Source Data file.

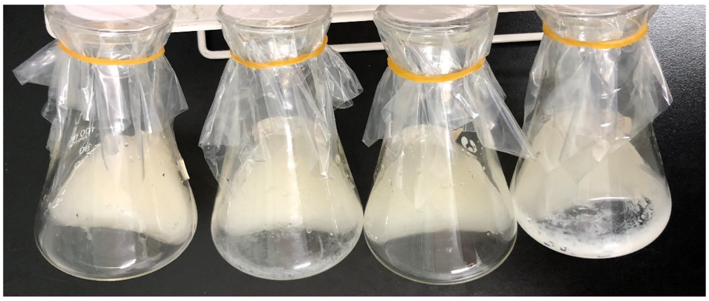

	WT	GTR1	GTR2	GTR3
faa1Δ, faa4Δ, pox1Δ, are2Δ	-	+	-	+
pah1Δ, dpp1Δ, lpp1Δ, are1Δ	-	-	+	+

Supplementary Figure 5. A picture shows that high levels of free fatty acids precipitated in flasks culture of GTR1 and GTR3 strains at 72 hours.

Supplementary Table 1. The primer design template for the Lightening GTR-CRISPR system (A2A2-KlU100)

PCR template	Primers	Primer Sequences
psgtRNA	Primer 1F	AAAGGTCTCAGATCNNNNNNNNNNNNNNNNNNNNGTTTTAGAGCTAGAAATAGCAAGTTA
	Primer 1R	AAAGGTCTCARRRRRRRRRRRRTGCGCAAGCCCGGAATCGAACCGGG
pKlURA100	Primer 2F	AAAGGTCTCANNNNNNNNNNNNGTTTTAGAGCTAGAAATAGCAAGTTAA
	Primer 2R	AAAGGTCTCARRRRRRRRRRRRGATCATTTATCTTTCACTGCGG
psgtRNA	Primer 3F	AAAGGTCTCANNNNNNNNNNNNGTTTTAGAGCTAGAAATAGCAAGTTAA
	Primer 3R	AAAGGTCTCTAAACRRRRRRRRRRRRRRRRRRRRTGCGCAAGCCCGGAATCGAACCGGG

Note: N means the forward DNA sequence, R mean the reverse DNA sequence. The letters in red represent for the 1st gRNA target, greens for the 2nd target, yellows for the 3rd target, and blues for the 4th target. The letters highlighted in brown and blue are ligated to the pCas plasmid. And letters highlighted in green or yellow are ligated with each other with same color. For the second and third targets, the split of 20 base pairs does not need to be evenly distributed.

Supplementary Table 2. Time calculation (days) of different systems for multiplexed CRISPR/Cas9 based gene editing systems.

CRISPR systems	Gene synthesis	Cas9 pre-transformation[b]	Helper plasmid[b]	gRNA plasmid construction[c]	Recovery after transformation	Yeast growth from plates	Total time spent
**Plasmid-required systems**							
HI-CRISPR[1]	3-5[a]	-	-	3-4	2	2	10-13
CRISPRm[2]	-	-	3-4	3-4	-	2	8-10
Csy4 based CRISPR[3]	-	3	3-4	3-4	-	2	11-13
CasEMBLR[4, 5]	-	3	3-4	3-4	-	2	11-13
CRISPR by Mans and Rossum *et al*[6]	-	3	-	3-4	-	2	8-9
GTR-CRISPR (This work)	-	-	-	3-4	1	2	6-7
**Cloning-free systems**							
CRISPR by Generoso *et al*[7]	-	-	-	-	-	2	2
CAM[8, 9]	-	3	-	-	-	2	5
Lightning GTR-CRISPR (This work)	-	-	-	-	1	2	3

[a]gBlocks gene fragments (124-750bp) services provided from IDTDNA(US) for 2-4 days shipped, which is 3-5 days arrived.

[bc]Calculation includes PCR, enzyme digestion and ligation, *E. coli* transformation and miniprep, and plasmid sequencing.

[b]For some methods, the helper plasmids have to be pre-built for construction the final gRNA plasmids.

Supplementary Note

DNA sequences for PCR amplification

psgtRNA

GTTTTAGAGCTAGAAATAGCAAGTTAAAATAAGGCTAGTCCGTTATCAACTTGAAAAAGT
GGCACCGAGTCGGTGCAAACAAGCGCAAGTGGTTTAGTGGTAAAATCCAACGTTGCCATC
GTTGGGCCCCCGGTTCGATTCCGGGCTTGCGCA

Note: Sequence in yellow: gRNA scaffold; sequence in dark: linker; sequence in purple: $tRNA^{Gly}$.

pSNRtRNA

GTTTTAGAGCTAGAAATAGCAAGTTAAAATAAGGCTAGTCCGTTATCAACTTGAAAAAGT
GGCACCGAGTCGGTGCTTTTTTTATTTTTTGTCACTATTGTTATGTAAAATGCCACCTCTGA
CAGTATGGAACGCAAACTTCTGTCTAGTGGATAACAGAATTTTTCTATGGCCAATTTAGGA
TCCACTAGTCTTTGAAAAGATAATGTATGATTATGCTTTCACTCATATTTATACAGAAACT
TGATGTTTTCTTTCGAGTATATACAAGGTGATTACATGTACGTTTGAAGTACAACTCTAGA
TTTTGTAGTGCCCTCTTGGGCTAGCGGTAAAGGTGCGCATTTTTTCACACCCTACAATGTTC
TGTTCAAAAGATTTTGGTCAAACGCTGTAGAAGTGAAAGTTGGTGCGCATGTTTCGGCGTT
CGAAACTTCTCCGCAGTGAAAGATAAATGATCTAGTTTTCACAAGAAAACAAGCGCAAGT
GGTTTAGTGGTAAAATCCAACGTTGCCATCGTTGGGCCCCCGGTTCGATTCCGGGCTTGCG
CA

Note: Sequence in yellow: gRNA scaffold; sequence in green: *SNR52* terminator; sequence in blue: *SNR52* promoter; sequence in purple: $tRNA^{Gly}$.

pScURA

GTTTTAGAGCTAGAAATAGCAAGTTAAAATAAGGCTAGTCCGTTATCAACTTGAAAAAGT
GGCACCGAGTCGGTGCTTTTTTTATTTTTTGTCACTATTGTTATGTAAAATGCCACCTCTGA
CAGTATGGAACGCAAACTTCTGTCTAGTGGATAACAGAATTTTTCTATGGCCAATTTAGGA
TCCCGCGAACCCAACTGCACAGAACAAAAACCTGCAGGAAACGAAGATAAATCATGTCG
AAAGCTACATATAAGGAACGTGCTGCTACTCATCCTAGTCCTGTTGCTGCCAAGCTATTTA
ATATCATGCACGAAAAGCAAACAAACTTGTGTGCTTCATTGGATGTTCGTACCACCAAGG
AATTACTGGAGTTAGTTGAAGCATTAGGTCCCAAAATTTGTTTACTAAAAACACATGTGGA
TATCTTGACTGATTTTTCCATGGAGGGCACAGTTAAGCCGCTAAAGGCATTATCCGCCAAG
TACAATTTTTTACTCTTCGAAGACAGAAAATTTGCTGACATTGGTAATACAGTCAAATTGC
AGTACTCTGCGGGTGTATACAGAATAGCAGAATGGGCAGACATTACGAATGCACACGGTG
TGGTGGGCCCAGGTATTGTTAGCGGTTTGAAGCAGGCGGCAGAAGAAGTAACAAAGGAA
CCTAGAGGCCTTTTGATGTTAGCAGAATTGTCATGCAAGGGCTCCCTATCTACTGGAGAAT
ATACTAAGGGTACTGTTGACATTGCGAAGAGCGACAAAGATTTTGTTATCGGCTTTATTGC
TCAAAGAGACATGGGTGGAAGAGATGAAGGTTACGATTGGTTGATTATGACACCCGGTGT
GGGTTTAGATGACAAGGGAGACGCATTGGGTCAACAGTATAGAACCGTGGATGATGTCGT
TTCTACAGGATCTGACATTATTATTGTTGGAAGAGGACTATTTGCAAAGGGAAGGGATGC
TAAGGTAGAGGGTGAACGTTACAGAAAAGCAGGCTGGGAAGCATATTTGAGAAGATGCG
GCCAGCAAAACTAAAAAACTGTATTATAAGTAAATGCATGTATACTAAACTCACAAATTA
GAGCTTCAATTTAATTATATCAGTTATTACCCTGTGTCTAGTCTTTGAAAAGATAATGTAT
GATTATGCTTTCACTCATATTTATACAGAAACTTGATGTTTTCTTTCGAGTATATACAAGGT
GATTACATGTACGTTTGAAGTACAACTCTAGATTTTGTAGTGCCCTCTTGGGCTAGCGGTA
AAGGTGCGCATTTTTTCACACCCTACAATGTTCTGTTCAAAAGATTTTGGTCAAACGCTGT
AGAAGTGAAAGTTGGTGCGCATGTTTCGGCGTTCGAAACTTCTCCGCAGTGAAAGATAAA
TGATCTAGTTTTCACAAGAAAACAAGCGCAAGTGGTTTAGTGGTAAAATCCAACGTTGCC
ATCGTTGGGCCCCCGGTTCGATTCCGGGCTTGCGCA

Note: Sequence in yellow: gRNA scaffold; sequence in green: *SNR52* terminator; sequence in red: *URA3* from *S. cerevisiae* (the sequences around it without color are its endogenous 50bp promoter and terminator); sequence in blue: *SNR52* promoter; sequence in purple: $tRNA^{Gly}$.

pKlURA300

GTTTTAGAGCTAGAAATAGCAAGTTAAAATAAGGCTAGTCCGTTATCAACTTGAAAAAGT
GGCACCGAGTCGGTGCTTTTTTTCTTTTTGTCCACCACCAAAATGTCAATTTTTTGCCAT
TTTCTAAATAGTTTTCTTCACTCTACAAGAGATATCACGATTGTTTGCTTTCGATATTGGAT
CCTCGGTACATAAATATATGTGATTCTGGGTAGAAGATCGGTCTGCATTGGATGGTGGTAA
CGCATTTTTTTACACACATTACTTGCCTCGAGCATCAAATGGTGGTTATTCGTGGATCTATA
TCACGTGATTTGCTTAAGAATTGTCGTTCATGGTGACACTTTTAGCTTTGACATGATTAAG
CTCATCTCAATTGATGTTATCTAAAGTCATTTCAACTATCTAAGATGTGGTTGTGATTGGG
CCATTTTGTGAAAGCCAGTACGCCAGCGTCAATACACTCCCGTCAATTAGTTGCACCATGT
CCACAAAATCATATACCAGTAGAGCTGAGACTCATGCAAGTCCGGTTGCATCGAAACTTT
TACGTTTAATGGATGAAAAGAAGACCAATTTGTGTGCTTCTCTTGACGTTCGTTCGACTGA
TGAGCTATTGAAACTTGTTGAAACGTTGGGTCCATACATTTGCCTTTTGAAAACACACGTT
GATATCTTGGATGATTTCAGTTATGAGGGTACTGTCGTTCCATTGAAAGCATTGGCAGAGA
AATACAAGTTCTTGATATTTGAGGACAGAAAATTCGCCGATATCGGTAACACAGTCAAAT
TACAATATACATCGGGCGTTTACCGTATCGCAGAATGGTCTGATATCACCAACGCCCACG
GGGTTACTGGTGCTGGTATTGTTGCTGGCTTGAAACAAGGTGCGCAAGAGGTCACCAAAG
AACCAAGGGGATTATTGATGCTTGCTGAATTGTCTTCCAAGGGTTCTCTAGCACACGGTGA
ATATACTAAGGGTACCGTTGATATTGCAAAGAGTGATAAAGATTTCGTTATTGGGTTCATT
GCTCAGAACGATATGGGAGGAAGAGAAGAAGGGTTTGATTGGCTAATCATGACCCCAGGT
GTAGGTTTAGACGACAAAGGCGATGCATTGGGTCAGCAGTACAGAACCGTCGACGAAGTT
GTAAGTGGTGGATCAGATATCATCATTGTTGGCAGAGGACTTTTCGCCAAGGGTAGAGAT
CCTAAGGTTGAAGGTGAAAGATACAGAAATGCTGGATGGGAAGCGTACCAAAAGAGAAT
CAGCGCTCCCCATTAATTATACAGGAAACTTAATAGAACAAATCACATATTTAATCTAATA
GCCACCTGCATTGGCACGGTGCAACACTACTTCAACTTCATCTTACAAAAAGATCACGTGA
TCTGTTGTATTGAACTGAAAATTTTTTGTTTGCTTCTCTCTCTCTCTCTTTCATTATGTGAGA
TTTAAAAACCAGAAACTACATCATCGAAAAAGTCTAGACTTTGAAAAGATAATGTATGAT
TATGCTTTCACTCATATTTATACAGAAACTTGATGTTTTCTTTCGAGTATATACAAGGTGAT
TACATGTACGTTTGAAGTACAACTCTAGATTTTGTAGTGCCCTCTTGGGCTAGCGGTAAAG
GTGCGCATTTTTTCACACCCTACAATGTTCTGTTCAAAAGATTTTGGTCAAACGCTGTAGA
AGTGAAAGTTGGTGCGCATGTTTCGGCGTTCGAAACTTCTCCGCAGTGAAAGATAAATGA
TCTAGTTTTCACAAGAAAACAAGCGCAAGTGGTTTAGTGGTAAAATCCAACGTTGCCATC
GTTGGGCCCCCGGTTCGATTCCGGGCTTGCGCA

Note: Sequence in yellow: gRNA scaffold; sequence in dark green: *SUF17* terminator; sequence in red: *URA3* from *Kluyveromyces lactis* (the sequences around it without color are its endogenous 300 bp promoter and terminator); sequence in blue: *SNR52* promoter; sequence in purple: $tRNA^{Gly}$.

pKlURA200

GTTTTAGAGCTAGAAATAGCAAGTTAAAATAAGGCTAGTCCGTTATCAACTTGAAAAAGT
GGCACCGAGTCGGTGCTTTTTTTCTTTTTTGTCCACCACCAAAATGTCAATTTTTTTGCCAT
TTTCTAAATAGTTTTCTTCACTCTACAAGAGATATCACGATTGTTTGCTTTCGATATTGGAT
CCGTGGTTATTCGTGGATCTATATCACGTGATTTGCTTAAGAATTGTCGTTCATGGTGACA
CTTTTAGCTTTGACATGATTAAGCTCATCTCAATTGATGTTATCTAAAGTCATTTCAACTAT
CTAAGATGTGGTTGTGATTGGGCCATTTTGTGAAAGCCAGTACGCCAGCGTCAATACACTC
CCGTCAATTAGTTGCACCATGTCCACAAAATCATATACCAGTAGAGCTGAGACTCATGCA
AGTCCGGTTGCATCGAAACTTTTACGTTTAATGGATGAAAAGAAGACCAATTTGTGTGCTT
CTCTTGACGTTCGTTCGACTGATGAGCTATTGAAACTTGTTGAAACGTTGGGTCCATACAT
TTGCCTTTTGAAAACACACGTTGATATCTTGGATGATTTCAGTTATGAGGGTACTGTCGTT
CCATTGAAAGCATTGGCAGAGAAATACAAGTTCTTGATATTTGAGGACAGAAAATTCGCC
GATATCGGTAACACAGTCAAATTACAATATACATCGGGCGTTTACCGTATCGCAGAATGG
TCTGATATCACCAACGCCCACGGGGTTACTGGTGCTGGTATTGTTGCTGGCTTGAAACAAG
GTGCGCAAGAGGTCACCAAAGAACCAAGGGGATTATTGATGCTTGCTGAATTGTCTTCCA
AGGGTTCTCTAGCACACGGTGAATATACTAAGGGTACCGTTGATATTGCAAAGAGTGATA
AAGATTTCGTTATTGGGTTCATTGCTCAGAACGATATGGGAGGAAGAGAAGAAGGGTTTG
ATTGGCTAATCATGACCCCAGGTGTAGGTTTAGACGACAAAGGCGATGCATTGGGTCAGC
AGTACAGAACCGTCGACGAAGTTGTAAGTGGTGGATCAGATATCATCATTGTTGGCAGAG
GACTTTTCGCCAAGGGTAGAGATCCTAAGGTTGAAGGTGAAAGATACAGAAATGCTGGAT
GGGAAGCGTACCAAAAGAGAATCAGCGCTCCCCATTAATTATACAGGAAACTTAATAGAA
CAAATCACATATTTAATCTAATAGCCACCTGCATTGGCACGGTGCAACACTACTTCAACTT
CATCTTACAAAAAGATCACGTGATCTGTTGTATTGAACTGAAAATTTTTTGTTTGCTTCTCT
CTCTCTCTCTTTCATTATGTGAGATTTAAAAACCAGAAACTACATCATCGAAAAAGTCTAG
ACTTTGAAAAGATAATGTATGATTATGCTTTCACTCATATTTATACAGAAACTTGATGTTTT
CTTTCGAGTATATACAAGGTGATTACATGTACGTTTGAAGTACAACTCTAGATTTTGTAGT
GCCCTCTTGGGCTAGCGGTAAAGGTGCGCATTTTTTCACACCCTACAATGTTCTGTTCAAA
AGATTTTGGTCAAACGCTGTAGAAGTGAAAGTTGGTGCGCATGTTTCGGCGTTCGAAACTT
CTCCGCAGTGAAAGATAAATGATCTAGTTTTCACAAGAAAACAAGCGCAAGTGGTTTAGT
GGTAAAATCCAACGTTGCCATCGTTGGCCCCCGGTTCGATTCCGGGCTTGCGCA

Note: Sequence in yellow: gRNA scaffold; sequence in dark green: *SUF17* terminator; sequence in red: *URA3* from *Kluyveromyces lactis* (the sequences around it without color are its endogenous 200 bp promoter and terminator); sequence in blue: *SNR52* promoter; sequence in purple: $tRNA^{Gly}$.

pKlURA100

GTTTTAGAGCTAGAAATAGCAAGTTAAAATAAGGCTAGTCCGTTATCAACTTGAAAAAGT
GGCACCGAGTCGGTGCTTTTTTTCTTTTTTGTCCACCACCAAAATGTCAATTTTTTTGCCAT
TTTCTAAATAGTTTTCTTCACTCTACAAGAGATATCACGATTGTTTGCTTTCGATATTGGAT
CCATCTAAAGTCATTTCAACTATCTAAGATGTGGTTGTGATTGGGCCATTTTGTGAAAGCC
AGTACGCCAGCGTCAATACACTCCCGTCAATTAGTTGCACCATGTCCACAAAATCATATAC
CAGTAGAGCTGAGACTCATGCAAGTCCGGTTGCATCGAAACTTTTACGTTTAATGGATGA
AAAGAAGACCAATTTGTGTGCTTCTCTTGACGTTCGTTCGACTGATGAGCTATTGAAACTT
GTTGAAACGTTGGGTCCATACATTTGCCTTTTGAAAACACACGTTGATATCTTGGATGATT
TCAGTTATGAGGGTACTGTCGTTCCATTGAAAGCATTGGCAGAGAAATACAAGTTCTTGAT
ATTTGAGGACAGAAAATTCGCCGATATCGGTAACACAGTCAAATTACAATATACATCGGG
CGTTTACCGTATCGCAGAATGGTCTGATATCACCAACGCCCACGGGGTTACTGGTGCTGGT
ATTGTTGCTGGCTTGAAACAAGGTGCGCAAGAGGTCACCAAAGAACCAAGGGGATTATTG
ATGCTTGCTGAATTGTCTTCCAAGGGTTCTCTAGCACACGGTGAATATACTAAGGGTACCG
TTGATATTGCAAAGAGTGATAAAGATTTCGTTATTGGGTTCATTGCTCAGAACGATATGGG
AGGAAGAGAAGAAGGGTTTGATTGGCTAATCATGACCCCAGGTGTAGGTTTAGACGACAA
AGGCGATGCATTGGGTCAGCAGTACAGAACCGTCGACGAAGTTGTAAGTGGTGGATCAGA
TATCATCATTGTTGGCAGAGGACTTTTCGCCAAGGGTAGAGATCCTAAGGTTGAAGGTGA
AAGATACAGAAATGCTGGATGGGAAGCGTACCAAAAGAGAATCAGCGCTCCCCATTAATT
ATACAGGAAACTTAATAGAACAAATCACATATTTAATCTAATAGCCACCTGCATTGGCAC
GGTGCAACACTACTTCAACTTCATCTTACAAAAAGATCACGTGATCTGTTGTATTGAACTG
AAAATTTTTTGTTTGCTTCTCTCTCTCTCTCTTTCATTATGTGAGATTTAAAAACCAGAAAC
TACATCATCGAAAAAGTCTAGACTTTGAAAAGATAATGTATGATTATGCTTTCACTCATAT
TTATACAGAAACTTGATGTTTTCTTTCGAGTATATACAAGGTGATTACATGTACGTTTGAA
GTACAACTCTAGATTTTGTAGTGCCCTCTTGGGCTAGCGGTAAAGGTGCGCATTTTTTCAC
ACCCTACAATGTTCTGTTCAAAAGATTTTGGTCAAACGCTGTAGAAGTGAAAGTTGGTGCG
CATGTTTCGGCGTTCGAAACTTCTCCGCAGTGAAAGATAAATGATCTAGTTTTCACAAGAA
AACAAGCGCAAGTGGTTTAGTGGTAAAATCCAACGTTGCCATCGTTGGGCCCCCGGTTCG
ATTCCGGGCTTGCGCA

Note: Sequence in yellow: gRNA scaffold; sequence in dark green: *SUF17* terminator; sequence in red: *URA3* from *Kluyveromyces lactis* (the sequences around it without color are its endogenous 100 bp promoter and terminator); sequence in blue: *SNR52* promoter; sequence in purple: $tRNA^{Gly}$.

pSCLEU

GTTTTAGAGCTAGAAATAGCAAGTTAAAATAAGGCTAGTCCGTTATCAACTTGAAAAAGT
GGCACCGAGTCGGTGCTTTTTTTCTTTTTTGTCCACCACCAAAATGTCAATTTTTTTGCCAT
TTTCTAAATAGTTTTCTTCACTCTACAAGAGATATCACGATTGTTTGCTTTCGATATTGGAT
CCAACTGTGGGAATACTCAGGTATCGTAAGATGCAAGAGTTCGAATCTCTTAGCAACCAT
TATTTTTTTCCTCAACATAACGAGAACACACAGGGGCGCTATCGCACAGAATCAAATTCG
ATGACTGGAAATTTTTTGTTAATTTCAGAGGTCGCCTGACGCATATACCTTTTTCAACTGA
AAAATTGGGAGAAAAAGGAAAGGTGAGAGCGCCGGAACCGGCTTTTCATATAGAATAGA
GAAGCGTTCATGACTAAATGCTTGCATCACAATACTTGAAGTTGACAATATTATTTAAGGA
CCTATTGTTTTTTCCAATAGGTGGTTAGCAATCGTCTTACTTTCTAACTTTTCTTACCTTTTA
CATTTCAGCAATATATATATATATATATTTCAAGGATATACCATTCTAATGTCTGCCCCTAAG
AAGATCGTCGTTTTGCCAGGTGACCACGTTGGTCAAGAAATCACAGCCGAAGCCATTAAG
GTTCTTAAAGCTATTTCTGATGTTCGTTCCAATGTCAAGTTCGATTTCGAAAATCATTTAAT
TGGTGGTGCTGCTATCGATGCTACAGGTGTTCCACTTCCAGATGAGGCGCTGGAAGCCTCC
AAGAAGGCTGATGCCGTTTTGTTAGGTGCTGTGGGTGGTCCTAAATGGGGTACCGGTAGT
GTTAGACCTGAACAAGGTTTACTAAAAATCCGTAAAGAACTTCAATTGTACGCCAACTTA
AGACCATGTAACTTTGCATCCGACTCTCTTTTAGACTTATCTCCAATCAAGCCACAATTTG
CTAAAGGTACTGACTTCGTTGTTGTCAGAGAATTAGTGGGAGGTATTTACTTTGGTAAGAG
AAAGGAAGACGATGGTGATGGTGTCGCTTGGGATAGTGAACAATACACCGTTCCAGAAGT
GCAAAGAATCACAAGAATGGCCGCTTTCATGGCCCTACAACATGAGCCACCATTGCCTAT
TTGGTCCTTGGATAAAGCTAATGTTTTGGCCTCTTCAAGATTATGGAGAAAAACTGTGGAG
GAAACCATCAAGAACGAATTCCCTACATTGAAGGTTCAACATCAATTGATTGATTCTGCCG
CCATGATCCTAGTTAAGAACCCAACCCACCTAAATGGTATTATAATCACCAGCAACATGTT
TGGTGATATCATCTCCGATGAAGCCTCCGTTATCCCAGGTTCCTTGGGTTTGTTGCCATCTG
CGTCCTTGGCCTCTTTGCCAGACAAGAACACCGCATTTGGTTTGTACGAACCATGCCACGG
TTCTGCTCCAGATTTGCCAAAGAATAAGGTCAACCCTATCGCCACTATCTTGTCTGCTGCA
ATGATGTTGAAATTGTCATTGAACTTGCCTGAAGAAGGTAAGGCCATTGAAGATGCAGTT
AAAAAGGTTTTGGATGCAGGTATCAGAACTGGTGATTTAGGTGGTTCCAACAGTACCACC
GAAGTCGGTGATGCTGTCGCCGAAGAAGTTAAGAAAATCCTTGCTTAAAAAGATTCTCTTT
TTTTATGATATTTGTACATAAACTTTATAAATGAAATTCATAATAGAAACGACACGAAATT
ACAAAATGGAATATGTTCATAGGGTAGACGAAACTATATACGCAATCTACATACATTTAT
CAAGAAGGAGAAAAAGGAGGATGTAAAGGAATACAGGTAAGCAAATTGATACTAATGGC
TCAACGGACTTTGAAAAGATAATGTATGATTATGCTTTCACTCATATTTATACAGAAACTT
GATGTTTTCTTTCGAGTATATACAAGGTGATTACATGTACGTTTGAAGTACAACTCTAGAT
TTTGTAGTGCCCTCTTGGGCTAGCGGTAAAGGTGCGCATTTTTTCACACCCTACAATGTTCT
GTTCAAAAGATTTGGTCAAACGCTGTAGAAGTGAAAGTTGGTGCGCATGTTTCGGCGTTC
GAAACTTCTCCGCAGTGAAAGATAAATGATCTAGTTTCACAAGAAAACAAGCGCAAGTG
GTTTAGTGGTAAAATCCAACGTTGCCATCGTTGGGCCCCCGGTTCGATTCCGGGCTTGCGC
A

Note: Sequence in yellow: gRNA scaffold; sequence in dark green: *SUF17* terminator; sequence in red: *LEU2* from *S. cerevisiae* (the sequences around it without color are its endogenous 408bp promoter and terminator); sequence in blue: *SNR52* promoter; sequence in purple: $tRNA^{Gly}$.

Full Plasmid Sequences for pCas vector:

```
LOCUS       Exported        8713 bp ds-DNA     circular SYN 16-AUG-2018
DEFINITION  synthetic circular DNA
ACCESSION   .
VERSION     .
KEYWORDS    pCas
SOURCE      synthetic DNA construct
  ORGANISM  recombinant plasmid
REFERENCE   1  (bases 1 to 8713)
  AUTHORS   Yueping Zhang
FEATURES             Location/Qualifiers
     source          1..8713
                     /organism="recombinant plasmid"
                     /mol_type="other DNA"
     rep_origin      2..1344
                     /label=2u ori
                     /note="yeast 2u plasmid origin of replication"
     promoter        1371..1475
                     /gene="bla"
                     /label=AmpR promoter
     CDS             1476..2336
                     /codon_start=1
                     /gene="bla"
                     /product="beta-lactamase"
                     /label=AmpR
                     /note="confers resistance to ampicillin, carbenicillin, and
                     related antibiotics"
                     /translation="MSIQHFRVALIPFFAAFCLPVFAHPETLVKVKDAEDQLGARVGYI
                     ELDLNSGKILESFRPEERFPMMSTFKVLLCGAVLSRIDAGQEQLGRRIHYSQNDLVEYS
                     PVTEKHLTDGMTVRELCSAAITMSDNTAANLLLTTIGGPKELTAFLHNMGDHVTRLDRW
                     EPELNEAIPNDERDTTMPVAMATTLRKLLTGELLTLASRQQLIDWMEADKVAGPLLRSA
                     LPAGWFIADKSGAGERGSRGIIAALGPDGKPSRIVVIYTTGSQATMDERNRQIAEIGAS
                     LIKHW"
     rep_origin      2507..3095
                     /direction=RIGHT
                     /label=ori
                     /note="high-copy-number ColE1/pMB1/pBR322/pUC origin of
                     replication"
     promoter        3108..3376
                     /label=SNR52 promoter
                     /note="promoter for the S. cerevisiae small nucleolar RNA
                     gene SNR52"
     CDS             complement(3384..3680)
```

/codon_start=1
/label=lacZ-alpha
/translation="MTMITPSAQLTLTKGNKSWRTSRGGPVPNSPYSESYYARSLAVVL
QRRDWENPGVTQLNRLAAHPPFASWRNSEEARTDRPSQQLRSLNGEWDAPCSG"

promoter complement(3724..3754)
/label=lac promoter
/note="promoter for the E. coli lac operon"

misc_RNA 3836..3907
/label=gRNA scaffold
/note="guide RNA scaffold for the Streptococcus pyogenes
CRISPR/Cas9 system"

terminator 3936..4123
/gene="S. cerevisiae ADH1"
/label=ADH1 terminator
/note="transcription terminator for the S. cerevisiae
alcohol dehydrogenase 1 (ADH1) gene"

CDS complement(4133..4153)
/codon_start=1
/product="nuclear localization signal of SV40 large T
antigen"
/label=SV40 NLS
/translation="PKKKRKV"

CDS complement(4157..8257)
/codon_start=1
/product="Cas9 (Csn1) endonuclease from the Streptococcus
pyogenes Type II CRISPR/Cas system"
/label=iCas9
/note="generates RNA-guided double strand breaks in DNA"
/translation="DKKYSIGLDIGTNSVGWAVITDEYKVPSKKFKVLGNTDRHSIKKN
LIGALLFDSGETAEATRLKRTARRRYTRRKNRICYLQEIFSNEMAKVDDSFFHRLEESF
LVEEDKKHERHPIFGNIVDEVAYHEKYPTIYHLRKKLVDSTYKADLRLIYLALAHMIKF
RGHFLIEGDLNPDNSDVDKLFIQLVQTYNQLFEENPINASGVDAKAILSARLSKSRRLE
NLIAQLPGEKKNGLFGNLIALSLGLTPNFKSNFDLAEDAKLQLSKDTYDDDLDNLLAQI
GDQYADLFLAAKNLSDAILLSDILRVNTEITKAPLSASMIKRYDEHHQDLTLLKALVRQ
QLPEKYKEIFFDQSKNGYAGYIDGGASQEEFYKFIKPILEKMDGTEELLVKLNREDLLR
KQRTFDNGSITHQIHLGELHAILRRQEDFYPFLKDNREKIEKILTFRIPYYVGPLARGN
SRFAWMTRKSEETITPWNFEEVVDKGASAQSFIERMTNFDKNLPNEKVLPKHSLLYEYF
TVYNELTKVKYVTEGMRKPAFLSGEQKKAIVDLLFKTNRKVTVKQLKEDYFKKIECFDS
VEISGVEDRFNASLGTYHDLLKIIKDKDFLDNEENEDILEDIVLTLTLFEDREMIEERL
KTYAHLFDDKVMKQLKRRRYTGWGRLSRKLINGIRDKQSGKTILDFLKSDGFANRNFMQ
LIHDDSLTFKEDIQKAQVSGQGDSLHEHIANLAGSPAIKKGILQTVKVVDELVKVMGRH
KPENIVIEMARENQTTQKGQKNSRERMKRIEEGIKELGSQILKEHPVENTQLQNEKLYL
YYLQNGRDMYVDQELDINRLSDYDVDHIVPQSFLKDDSIDNKVLTRSDKNRGKSDNVPS
EEVVKKMKNYWRQLLNAKLITQRKFDNLTKAERGGLSELDKAGFIKRQLVETRQITKHV

AQILDSRMNTKYDENDKLIREVKVITLKSKLVSDFRKDFQFYKVREINNYHHAHDAYLN
AVVGTALIKKYPKLESEFVYGDYKVYDVRKMIAKSEQEIGKATAKYFFYSNIMNFFKTE
ITLANGEIRKRPLIETNGETGEIVWDKGRDFATVRKVLSMPQVNIVKKTEVQTGGFSKE
SILPKRNSDKLIARKKDWDPKKYGGFDSPTVAYSVLVVAKVEKGKSKKLKSVKELLGIT
IMERSSFEKNPIDFLEAKGYKEVKKDLIIKLPKYSLFELENGRKRMLASAGELQKGNEL
ALPSKYVNFLYLASHYEKLKGSPEDNEQKQLFVEQHKHYLDEIIEQISEFSKRVILADA
NLDKVLSAYNKHRDKPIREQAENIIHLFTLTNLGAPAAFKYFDTTIDRKRYTSTKEVLD
ATLIHQSITGLYETRIDLSQLGGD"

CDS complement(8258..8278)
/codon_start=1
/product="nuclear localization signal of SV40 large T
antigen"
/label=SV40 NLS
/translation="PKKKRKV"

CDS complement(8282..8305)
/codon_start=1
/product="FLAG(R) epitope tag, followed by an enterokinase
cleavage site"
/label=FLAG
/translation="DYKDDDDK"

misc_feature complement(8309..8709)
/label=TEF1 promoter

ORIGIN

1 tgatccaata tcaaaggaaa tgatagcatt gaaggatgag actaatccaa ttgaggagtg
61 gcagcatata gaacagctaa agggtagtgc tgaaggaagc atacgatacc ccgcatggaa
121 tgggataata tcacaggagg tactagacta cctttcatcc tacataaata gacgcatata
181 agtacgcatt taagcataaa cacgcactat gccgttcttc tcatgtatat atatatacag
241 gcaacacgca gatataggtg cgacgtgaac agtgagctgt atgtgcgcag ctcgcgttgc
301 attttcggaa gcgctcgttt tcggaaacgc tttgaagttc ctattccgaa gttcctattc
361 tctagaaagt ataggaactt cagagcgctt ttgaaaacca aaagcgctct gaagacgcac
421 tttcaaaaaa ccaaaaacgc accggactgt aacgagctac taaaatattg cgaataccgc
481 ttccacaaac attgctcaaa agtatctctt tgctatatat ctctgtgcta tatccctata
541 taacctaccc atccaccttt cgctccttga acttgcatct aaactcgacc tctacatttt
601 ttatgtttat ctctagtatt actctttaga caaaaaaatt gtagtaagaa ctattcatag
661 agtgaatcga aaacaatacg aaaatgtaaa catttcctat acgtagtata tagagacaaa
721 atagaagaaa ccgttcataa ttttctgacc aatgaagaat catcaacgct atcactttct
781 gttcacaaag tatgcgcaat ccacatcggt atagaatata atcggggatg cctttatctt
841 gaaaaaatgc acccgcagct tcgctagtaa tcagtaaacg cgggaagtgg agtcaggctt
901 tttttatgga agagaaaata gacaccaaag tagccttctt ctaaccttaa cggacctaca
961 gtgcaaaaag ttatcaagag actgcattat agagcgcaca aaggagaaaa aaagtaatct
1021 aagatgcttt gttagaaaaa tagcgctctc gggatgcatt tttgtagaac aaaaaagaag
1081 tatagattct ttgttggtaa aatagcgctc tcgcgttgca tttctgttct gtaaaaatgc
1141 agctcagatt ctttgtttga aaaattagcg ctctcgcgtt gcatttttgt tttacaaaaa
1201 tgaagcacag attcttcgtt ggtaaaatag cgctttcgcg ttgcatttct gttctgtaaa

1261 aatgcagctc agattctttg tttgaaaaat tagcgctctc gcgttgcatt tttgttctac
1321 aaaatgaagc acagatgctt cgttcaggtg gcacttttcg gggaaatgtg cgcggaaccc
1381 ctatttgttt atttttctaa atacattcaa atatgtatcc gctcatgaga caataaccct
1441 gataaatgct tcaataatat tgaaaaagga agagtatgag tattcaacat ttccgtgtcg
1501 cccttattcc cttttttgcg gcattttgcc ttcctgtttt tgctcaccca gaaacgctgg
1561 tgaaagtaaa agatgctgaa gatcagttgg gtgcacgagt gggttacatc gaactggatc
1621 tcaacagcgg taagatcctt gagagttttc gccccgaaga acgttttcca atgatgagca
1681 cttttaaagt tctgctatgt ggcgcggtat tatcccgtat tgacgccggg caagagcaac
1741 tcggtcgccg catacactat tctcagaatg acttggttga gtactcacca gtcacagaaa
1801 agcatcttac ggatggcatg acagtaagag aattatgcag tgctgccata accatgagtg
1861 ataacactgc ggccaactta cttctgacaa cgatcggagg accgaaggag ctaaccgctt
1921 ttttgcacaa catgggggat catgtaactc gccttgatcg ttgggaaccg gagctgaatg
1981 aagccatacc aaacgacgag cgtgacacca cgatgcctgt agcaatggca acaacgttgc
2041 gcaaactatt aactggcgaa ctacttactc tagcttcccg gcaacaatta atagactgga
2101 tggaggcgga taaagttgca ggaccacttc tgcgctcggc ccttccggct ggctggttta
2161 ttgctgataa atctggagcc ggtgagcgtg ggagccgcgg tatcattgca gcactggggc
2221 cagatggtaa gccctcccgt atcgtagtta tctacacgac ggggagtcag gcaactatgg
2281 atgaacgaaa tagacagatc gctgagatag gtgcctcact gattaagcat tggtaactgt
2341 cagaccaagt ttactcatat atactttaga ttgatttaaa acttcatttt taatttaaaa
2401 ggatctaggt gaagatcctt tttgataatc tcatgaccaa aatcccttaa cgtgagtttt
2461 cgttccactg agcgtcagac cccgtagaaa agatcaaagg atcttcttga gatccttttt
2521 ttctgcgcgt aatctgctgc ttgcaaacaa aaaaaccacc gctaccagcg gtggtttgtt
2581 tgccggatca agagctacca actctttttc cgaaggtaac tggcttcagc agagcgcaga
2641 taccaaatac tgttcttcta gtgtagccgt agttaggcca ccacttcaag aactctgtag
2701 caccgcctac atacctcgct ctgctaatcc tgttaccagt ggctgctgcc agtggcgata
2761 agtcgtgtct taccgggttg gactcaagac gatagttacc ggataaggcg cagcggtcgg
2821 gctgaacggg gggttcgtgc acacagccca gcttggagcg aacgacctac accgaactga
2881 gatacctaca gcgtgagcta tgagaaagcg ccacgcttcc cgaagggaga aaggcggaca
2941 ggtatccggt aagcggcagg gtcggaacag gagagcgcac gagggagctt ccagggggaa
3001 acgcctggta tctttatagt cctgtcgggt ttcgccacct ctgacttgag cgtcgatttt
3061 tgtgatgctc gtcagggggg cggagcctat ggaaagcggc cgcacaatct ttgaaaagat
3121 aatgtatgat tatgctttca ctcatattta tacagaaact tgatgttttc tttcgagtat
3181 atacaaggtg attacatgta cgtttgaagt acaactctag attttgtagt gccctcttgg
3241 gctagcggta aaggtgcgca tttttcaca ccctacaatg ttctgttcaa aagattttgg
3301 tcaaacgctg tagaagtgaa agttggtgcg catgtttcgg cgttcgaaac ttctccgcag
3361 tgaaagataa atgatcagag acctcagccg ctacagggcg cgtcccattc gccattcagg
3421 ctgcgcaact gttgggaagg gcgatcggtg cgggcctctt cgctattacg ccagctggcg
3481 aaagggggat gtgctgcaag gcgattaagt tgggtaacgc cagggttttc ccagtcacga
3541 cgttgtaaaa cgacggccag tgagcgcgcg taatacgact cactataggg cgaattgggt
3601 acaggaccac cacggctcgt gcgccagctt ttgttccctt tagtgagggt taattgcgcg
3661 cttggcgtaa tcatggtcat agctgtttcc tgtgtgaaat tgttatccgc tcacaattcc
3721 acacaacata cgagccggaa gcataaagtg taaagcctgg ggtgcctaat gagtgagcta
3781 actcacatta attgcgttgc gctcactgcc cgctttccac cggtggtctc tgttttagag
3841 ctagaaatag caagttaaaa taaggctagt ccgttatcaa cttgaaaaag tggcaccgag

3901 tcggtgcttt ttttattttt tgtcactatt gcatgccggt agaggtgtgg tcaataagag
3961 cgacctcatg ctatacctga gaaagcaacc tgacctacag gaaagagtta ctcaagaata
4021 agaattttcg ttttaaaacc taagagtcac tttaaaattt gtatacactt atttttttta
4081 taacttattt aataataaaa atcataaatc ataagaaatt cgcctcgagt cagacctttc
4141 tcttcttttt tggagggtca cctcctagct gactcaaatc aatgcgtgtt tcataaagac
4201 cagtgatgga ttgatggata agagtggcat ctaaaacttc ttttgtagac gtatatcgtt
4261 tacgatcaat tgttgtatca aaatatttaa aagcagcggg agctccaaga ttcgtcaacg
4321 taaataaatg aataatattt tctgcttgtt cacgtattgg tttgtctcta tgtttgttat
4381 atgcactaag aactttatct aaattggcat ctgctaaaat aacacgctta gaaaattcac
4441 tgatttgctc aataatctca tctaaataat gcttatgctg ctccacaaac aattgttttt
4501 gttcgttatc ttctggacta cccttcaact tttcataatg actagctaaa tataaaaaat
4561 tcacatattt gcttggcaga gccagctcat ttcctttttg taattctccg gcactagcca
4621 gcatccgttt acgaccgttt tctaactcaa aaagactata tttaggtagt ttaatgatta
4681 agtctttttt aacttcctta tatcctttag cttctaaaaa gtcaatcgga tttttttcaa
4741 aggaacttct ttccataatt gtgatcccta gtaactcttt aacggatttt aacttcttcg
4801 atttcccttt ttccacctta gcaaccacta ggactgaata agctaccgtt ggactatcaa
4861 aaccaccata ttttttggga tcccagtctt ttttacgagc aataagcttg tccgaatttc
4921 tttttggtaa aattgactcc ttggagaatc cgcctgtctg tacttctgtt ttcttgacaa
4981 tattgacttg gggcatggac aatactttgc gcactgtggc aaaatctcgc cctttatccc
5041 agacaatttc tccagtttcc ccattagttt cgattagagg gcgtttgcga atctctccat
5101 ttgcaagtgt aatttctgtt ttgaagaagt tcatgatatt agagtaaaag aaatattttg
5161 cggttgcttt gcctatttct tgctcagact tagcaatcat tttacgaaca tcataaactt
5221 tataatcacc atagacaaac tccgattcaa gttttggata tttcttaatc aaagcagttc
5281 caacgacggc atttagatac gcatcatggg catgatggta attgttaatc tcacgtactt
5341 tatagaattg gaaatctttt cggaagtcag aaactaattt agattttaag gtaatcactt
5401 taacctctcg aataagttta tcattttcat cgtatttagt attcatgcga ctatccaaaa
5461 tttgtgccac atgcttagtg atttggcgag tttcaaccaa ttggcgtttg ataaaaccag
5521 ctttatcaag ttcactcaaa cctccacgtt cagctttcgt taaattatca aacttacgtt
5581 gagtgattaa cttggcgttt agaagttgtc tccaatagtt tttcatcttt ttgactactt
5641 cttcacttgg aacgttatcc gatttaccac gatttttatc agaacgcgtt aagaccttat
5701 tgtctattga atcgtcttta aggaaacttt gtggaacaat gtgatcgaca tcataatcac
5761 ttaaacgatt aatatctaat tcttggtcca catacatgtc tcttccattt tggagataat
5821 agagatagag cttttcattt tgcaattgag tattttcaac aggatgctct ttaagaatct
5881 gacttcctaa ttctttgata ccttcttcga ttcgtttcat acgctctcgc gaatttttct
5941 ggcccttttg agttgtctga ttttcacgtg ccatttcaat aacgatattt tctggcttat
6001 gccgccccat tactttgacc aattcatcaa caacttttac agtctgtaaa atacctttttt
6061 taatagcagg gctaccagct aaatttgcaa tatgttcatg taaactatcg ccttgtccag
6121 acacttgtgc tttttgaatg tcttctttaa atgtcaaact atcatcatgg atcagctgca
6181 taaaattgcg attggcaaaa ccatctgatt tcaaaaaatc taatattgtt ttgccagatt
6241 gcttatccct aataccatta atcaattttc gagacaaacg tccccaacca gtataacggc
6301 gacgtttaag ctgtttcatc accttatcat caaagaggtg agcatatgtt ttaagtcttt
6361 cctcaatcat ctccctatct tcaaataagg tcaatgttaa aacaatatcc tctaagatat
6421 cttcattttc ttcattatcc aaaaaatctt tatctttaat aatttttagc aaatcatggt
6481 aggtacctaa tgaagcatta aatctatctt caactcctga aatttcaaca ctatcaaaac

6541 attctatttt tttgaaataa tcttctttta attgcttaac ggttactttt cgatttgttt

6601 tgaagagtaa atcaacaatg gctttcttct gttcacctga aagaaatgct ggttttcgca

6661 ttccttcagt aacatatttg acctttgtca attcgttata aaccgtaaaa tactcataaa

6721 gcaaactatg ttttggtagt actttttcat ttggaagatt tttatcaaag tttgtcatgc

6781 gttcaataaa tgattgagct gaagcacctt tatcgacaac ttcttcaaaa ttccatgggg

6841 taattgtttc ttcagacttc cgagtcatcc atgcaaaacg actattgcca cgcgccaatg

6901 gaccaacata ataaggaatt cgaaaagtca agattttttc aatcttctca cgattgtctt

6961 ttaaaaatgg ataaaagtct tcttgtcttc tcaaaatagc atgcagctca cccaagtgaa

7021 tttgatgggt aatagagccg ttgtcaaagg tccgttgctt gcgcagcaaa tcttcacgat

7081 ttagtttcac caataattcc tcagtaccat ccattttttc taaaattggt ttgataaatt

7141 tataaaattc ttcttggcta gctcccccat caatataacc tgcatatccg ttttttgatt

7201 gatcaaaaaa gatttcttta tacttttctg gaagttgttg tcgaactaaa gcttttaaaa

7261 gagtcaagtc ttgatgatgt tcatcgtagc gtttaatcat tgaagctgat aggggagcct

7321 tagttatttc agtatttact cttaggatat ctgaaagtaa aatagcatct gataaattct

7381 tagctgccaa aaacaaatca gcatattgat ctccaatttg cgccaataaa ttatctaaat

7441 catcatcgta agtatctttt gaaagctgta atttagcatc ttctgccaaa tcaaaatttg

7501 atttaaaatt aggggtcaaa cccaatgaca aagcaatgag attcccaaat aagccatttt

7561 tcttctcacc ggggagctga gcaatgagat tttctaatcg tcttgattta ctcaatcgtg

7621 cagaaagaat cgctttagca tctactccac ttgcgttaat agggttttct tcaaataatt

7681 gattgtaggt ttgtaccaac tggataaata gtttgtccac atcactatta tcaggattta

7741 aatctccctc aatcaaaaaa tgaccacgaa acttaatcat atgcgctaag gccaaataga

7801 ttaagcgcaa atccgcttta taagtagaat ctaccaattt ttttcgcaga tgatagatag

7861 ttggatattt ctcatgataa gcaacttcat ctactatatt tccaaaaata ggatgacgtt

7921 catgcttctt gtcttcttcc accaaaaaag actcttcaag tcgatgaaag aaactatcat

7981 ctactttcgc catctcattt gaaaaaatct cctgtagata acaaatacga ttcttccgac

8041 gtgtatacct tctacgagct gtccgtttga gacgagtcgc ttccgctgtc tctccactgt

8101 caaataaaag agcccctata agattttttt tgatactgtg gcggtctgta tttcccagaa

8161 ccttgaactt tttagacgga accttatatt catcagtgat caccgcccat ccgacgctat

8221 ttgtgccgat atctaagcct attgagtatt tcttatcgac ctttctcttc ttttttggag

8281 gtttgtcatc gtcatcttta taatccatct agaaaactta gattagattg ctatgctttc

8341 tttctaatga gcaagaagta aaaaaagttg taatagaaca agaaaaatga aactgaaact

8401 tgagaaattg aagaccgttt attaacttaa atatcaatgg gaggtcatcg aaagagaaaa

8461 aaatcaaaaa aaaaattttc aagaaaaaga aacgtgataa aaatttttat tgcctttttc

8521 gacgaagaaa aagaaacgag gcggcctctt ttttcttttc caaaccttta gtacgggtaa

8581 ttaacgacac cctagaggaa gaaagagggg aaatttagta tgctgtgctt gggtgttttg

8641 aagtggtacg gcgatgcgcg gagtccgaga aaatctggaa gagtaaaaaa ggagtagaaa

8701 cattttgaag atc

//

References

1. Bao Z*, et al.* Homology-integrated CRISPR-Cas (HI-CRISPR) system for one-step multigene disruption in Saccharomyces cerevisiae. *ACS Synth Biol* **4**, 585-594 (2015).

2. Ryan OW*, et al.* Selection of chromosomal DNA libraries using a multiplex CRISPR system. *Elife* **3**, e03703 (2014).

3. Ferreira R, Skrekas C, Nielsen J, David F. Multiplexed CRISPR/Cas9 Genome Editing and Gene Regulation Using Csy4 in *Saccharomyces cerevisiae*. *ACS Synth Biol* **7**, 10-15 (2018).

4. Jakočiūnas T*, et al.* Multiplex metabolic pathway engineering using CRISPR/Cas9 in *Saccharomyces cerevisiae*. *Metab Eng* **28**, 213-222 (2015).

5. Jakočiūnas T*, et al.* CasEMBLR: Cas9-Facilitated Multiloci Genomic Integration of in Vivo Assembled DNA Parts in *Saccharomyces cerevisiae*. *ACS Synth Biol* **4**, 1226-1234 (2015).

6. Mans R*, et al.* CRISPR/Cas9: a molecular Swiss army knife for simultaneous introduction of multiple genetic modifications in Saccharomyces cerevisiae. *FEMS Yeast Res* **15**, fov004 (2015).

7. Generoso WC, Gottardi M, Oreb M, Boles E. Simplified CRISPR-Cas genome editing for Saccharomyces cerevisiae. *J Microbiol Methods* **127**, 203-205 (2016).

8. Horwitz AA*, et al.* Efficient Multiplexed Integration of Synergistic Alleles and Metabolic Pathways in Yeasts via CRISPR-Cas. *Cell Syst* **1**, 88-96 (2015).

9. Walter JM, Chandran SS, Horwitz AA. CRISPR-Cas-Assisted Multiplexing (CAM): Simple Same-Day Multi-Locus Engineering in Yeast. *J Cell Physiol* **231**, 2563-2569 (2016).
