# Supplementary material for: A gRNA-tRNA array for CRISPR-Cas9 based rapid multiplexed genome editing in Saccharomyces cerevisiae
Source: Nat Commun. 2019 Mar 5;10:1053. doi: 10.1038/s41467-019-09005-3 (PMC6400946; doi:10.1038/s41467-019-09005-3)
Supplement: Supplementary file 3 — Description of Additional Supplementary Files [file 41467_2019_9005_MOESM3_ESM.pdf]

Title: Supplementary data 1

Description: The strain genotypes, gRNA sequences, and primer sequences used in this paper are provided.
